# Job Crafting Profiles Among Chinese Generation Z New Nurses: The Role of Innovative Behavior and Management Implications

**DOI:** 10.1155/jonm/7889163

**Published:** 2026-08-30

**Authors:** Bo Zhu, Yang Dong, Boyi Li, Mei Zhou, Li Xu, Mingyu Zhang, Hongtao Guo, Honghong Wang

**Affiliations:** ^1^ School of Medical Humanities, Henan Medical University, Xinxiang, China; ^2^ Xiangya School of Nursing, Central South University, Changsha, China, csu.edu.cn; ^3^ School of Nursing, Inner Mongolia Medical University, Hohhot, China, immu.edu.cn; ^4^ Department of NICU, Affiliated Hospital of Inner Mongolia Medical University, Hohhot, China, nmgfy.com; ^5^ Department of Nursing, Affiliated Hospital of Inner Mongolia Medical University, Hohhot, China, nmgfy.com

**Keywords:** innovative behavior, job crafting, latent profile analysis, new nurses

## Abstract

**Aims:**

To identify the latent profiles of Chinese Generation Z new nurses′ job crafting, explore their demographic characteristics, and examine the predictive role of innovative behaviors in distinct job crafting profiles.

**Background:**

New nurses experience high levels of stress and turnover intention during early career transition, highlighting the importance of proactive work behaviors. Although job crafting and innovative behavior are both considered important for professional development and workforce sustainability, evidence on their association and heterogeneity among new nurses remains limited.

**Methods:**

This cross‐sectional study was conducted using convenience sampling among new nurses from three tertiary hospitals in Midwestern China between March and May 2023. Participants completed the Innovative Behavior Scale, the Job Crafting Scale, and a demographic questionnaire. Latent profile analysis (LPA) was applied to identify potential subgroups of job crafting among new nurses. LPA was conducted using Mplus 8.3, while descriptive statistics, chi‐square tests, analysis of variance, and multinomial logistic regression were performed using SPSS 27.0.

**Results:**

Three job crafting profiles were identified: “low job crafting profile” (43.3%), “moderate job crafting profile” (47.5%), and “high job crafting profile” (9.2%). Significant differences were observed in age, marital status, educational attainment, and innovative behavior (*P*< 0.05). Multinomial logistic regression further indicated that innovative behavior was a significant predictor of profile membership (*P*< 0.05).

**Conclusion:**

Heterogeneity in job crafting among Chinese Generation Z new nurses and the key role of innovative behavior in shaping job crafting patterns are evident, with implications for targeted managerial strategies to support professional development.

**Implications for Nursing Management:**

Practical implications include tailoring management strategies to each profile: supporting foundational skills and psychological safety for nurses in the low job crafting profile, promoting gradual task enhancement and professional confidence‐building for nurses in the Moderate job crafting profile, and sustaining empowerment and leadership development among nurses in the high job crafting profile.

## 1. Introduction

The nursing profession not only fulfills essential professional roles but also embodies significant social value. It encompasses comprehensive health promotion, which includes clinical support, compassionate care, and health education, while also extending into emerging areas to address diverse health needs [1]. The growth and development of new nurses, as the emerging force within the nursing workforce, are directly linked to the future of the nursing profession and the quality of the healthcare delivery system. Currently, there is no universally accepted definition of novice nurses, either domestically or internationally. Based on prior literature, this study defined “new nurses” as registered nurses with less than 3 years of total clinical nursing experience since initial licensure [2–5]. It is worth noting that the current new nurse group is predominantly composed of Generation Z nurses. Although the birth‐year range of Generation Z has not been uniformly defined in the literature, many studies place this cohort between the mid‐1990s and early 2010s. Based on a synthesis of relevant national and international studies, this study defines Generation Z as individuals born between 1995 and 2010 [6–10]. As digital natives, they are characterized by active thinking, strong receptivity to emerging technologies, and a capacity to introduce innovative perspectives into traditional nursing teams [11]. They possess a strong sense of individuality, a deep desire for work autonomy, and a need for self‐realization, demonstrating a willingness to take the initiative in contributing to their work [6].

Despite these advantages, current evidence suggests that the turnover intention among Generation Z nurses remains high and continues to increase [12, 13], primarily due to excessive occupational stress. A recent meta‐analysis synthesizing evidence from eight countries reported a pooled turnover intention among new nurses ranged from 27% to 46%, with high rates observed in Western regions (46%) compared to Asian contexts (29%) [14]. The pressures faced by new nurses primarily stem from three sources. First, heavy workloads and frequent night shifts contribute to fatigue, which in turn increases the risk of physical and mental health problems [15]. Second, limited clinical experience and insufficient coping abilities in complex situations often induce “transition shock”, leading to self‐doubt and heightened anxiety [16]. Third, challenges in workplace adaptation arise from inadequate social support and difficulties in interpersonal relationships, along with a diminished sense of professional identity and belonging [17, 18]. In addition, the work environment has been shown to significantly influence nurses’ professional quality of life [19]. Collectively, these factors pose significant challenges to the career stability of new nurses. Consequently, establishing a supportive environment during the initial phase of a new nurse’s career has emerged as a critical issue in nursing management practice.

After Wrzesniewski and Dutton first introduced the concept of job crafting in 2001, researchers began to explore its applications in organizational settings. As a significant notion within positive organizational behavior, job crafting transcends the limitations of “passive adaptation” inherent in traditional job design by emphasizing employees’ individual initiative and offering theoretical and practical implications for nursing management. Its theoretical foundation centers on empowering employees to take a leading role in their career development, encouraging them to actively modify their work tasks, relationships, and cognitive frameworks [20]. For new nurses experiencing professional transition, job crafting may serve as an effective strategy for mitigating transition‐related stress and reducing turnover. Existing research indicates that by proactively enhancing learning resources, optimizing work processes, or adjusting cognitive perspectives, nurses can markedly improve their initiative and sense of value in their roles, thereby reinforcing their professional identity and job satisfaction [21]. The positive self‐regulation facilitates clinical adaptation, supports personal growth, and contributes to the delivery of more humanistic and higher‐quality nursing care [22]. This is further supported by recent international evidence indicating that nurses’ psychological resources are positively associated with caring behaviors and care quality [23]. Moreover, job crafting aligns closely with Generation Z nurses’ pursuit of autonomy and personal value, enabling a shift from passive task execution to proactive role optimization.

From a theoretical perspective, the Job Demands–Resources (JD‐R) theory provides a valuable framework for understanding proactive work behaviors among new nurses [24]. The nursing work environment is characterized by high job demands, including workload, emotional labor, and role ambiguity, which may impair health when insufficiently buffered by resources. In contrast, job resources support motivation and positive work outcomes. Beyond contextual resources, the JD‐R framework also highlights the role of personal resources in shaping employees’ work‐related outcomes. In nursing populations, recent empirical studies have shown that individual characteristics are positively associated with adaptive work‐related outcomes, including proactive and self‐initiated work behaviors in clinical settings [25, 26]. These findings further support nurses’ proactive adjustment of work behaviors and resources. Recent extensions of the JD‐R theory incorporate perspectives on self‐regulation, suggesting that when confronted with elevated job demands and limited resources, employees may engage in proactive self‐regulatory strategies to manage those demands and maintain motivation and performance in high‐demand contexts, particularly during transitions or periods of role adjustment [27]. Through such proactive regulation, employees can optimize structural and social resources, adjust task boundaries, and derive meaning from work. Within this framework, job crafting and innovative behavior can be conceptualized as potential adaptive strategies that may assist new nurses in navigating workplace challenges and supporting their professional development.

The definition of personal innovative behavior applied in the medical field can be summarized as the ability of nurses to actively generate, adopt, and implement new methods, technologies, theories, or approaches to improve nursing care quality, promote health, prevent diseases, and enhance existing nursing practices [28, 29]. Nurses’ innovative behavior yields broad benefits across the healthcare system. At the organizational level, innovation enhances resource utilization and improves service quality and efficiency through the adoption of new technologies and optimized processes [30]. For patients, innovation directly enhances care quality and safety by providing more effective, personalized solutions for clinical needs, thereby improving patient experiences and treatment outcomes [31]. For nurses, innovation strengthens professional autonomy and fosters a stronger sense of professional value. When innovative ideas are recognized and implemented, nurses experience greater accomplishment and psychological fulfillment, which further reinforces continuous learning and professional growth [32]. This positive cycle enhances professional identity, particularly among Generation Z nurses whose strong desire for self‐realization and autonomy makes innovative practice a meaningful pathway for career fulfillment.

Previous research suggests a close association between new nurses’ job crafting and their innovative behavior [33]. However, existing nursing studies have largely adopted variable‐centered approaches, which may overlook the potential heterogeneity in how individuals combine different job crafting behaviors. In particular, few studies have applied person‐centered methods to examine the heterogeneity of job crafting among Generation Z new nurses or to explore the role of innovative behavior across different job crafting profiles. The JD‐R theory provides a useful framework for understanding proactive work behaviors among new nurses. Within this framework, job crafting and innovative behavior may serve as adaptive strategies for managing job demands and resources. Therefore, this study aimed to identify latent profiles of job crafting among Chinese Generation Z new nurses and to examine whether innovative behavior predicts membership in different job crafting profiles. Based on the theoretical framework and prior literature, the following hypotheses were proposed:

• H1: Distinct latent profiles of job crafting exist among Chinese Generation Z new nurses.
• H2: Innovative behavior differs significantly across the identified job crafting profiles.
• H3: Innovative behavior significantly predicts membership in different job crafting profiles after controlling for demographic variables.

Building on this premise, this study used latent profile analysis (LPA) to identify distinct job crafting profiles among new nurses and to examine how innovative behavior and individual characteristics influence these profiles. Meaningful differences in demographic characteristics and innovative behavior across different job crafting profiles may help nursing managers better understand the heterogeneity of work behaviors among new nurses.

## 2. Methods

### 2.1. Research Design

This study adopted a cross‐sectional design, collecting data from participants at a single time point to investigate the relationships between the variables of interest. This study followed the Strengthening the Reporting of Observational Studies in Epidemiology (STROBE) guidelines for cross‐sectional studies. In accordance with these recommendations, we provided detailed descriptions of the study setting and participants, eligibility criteria, sample size estimation, variables and measurement instruments, potential sources of bias, and statistical analysis methods to ensure transparency and reproducibility.

### 2.2. Settings and Participants

This study was conducted at three tertiary‐level general hospitals in Midwestern China between March and May 2023. These hospitals were selected using a convenience sampling method, primarily based on accessibility and their willingness to participate. Eligible participants were registered clinical nurses currently employed at these institutions.

The inclusion criteria for this study were (1) being a registered nurse employed by the hospital and (2) having less than 3 years of total clinical nursing experience since initial licensure. The exclusion criteria included (1) nurses who were not on duty during the data collection period (e.g., due to sick leave, maternity leave, or other personal reasons) and (2) those who were concurrently participating in other research projects.

Participant recruitment was conducted via departmental announcements and internal communication platforms. All nurses who met the inclusion criteria were invited to complete a structured electronic questionnaire during the study period.

### 2.3. Sample Size

The required sample size for LPA depends on several factors, including the number of indicators, the expected number of profiles, the degree of profile separation, and the size of the smallest latent class. Previous methodological and simulation studies suggest that a sample size of approximately 300∼500 is generally sufficient to obtain stable and reliable class solutions. In addition, it has been recommended that each latent class should ideally comprise at least 5% of the total sample to ensure sufficient class stability and interpretability [34–39].

A total of 405 questionnaires were distributed. After eligibility and data‐quality screening, 65 questionnaires were excluded for the following reasons: 18 respondents reported more than 3 years of work experience and therefore did not meet the inclusion criteria for new nurses; 21 questionnaires contained substantial missing data; 9 showed logical inconsistencies in responses; 8 had extremely short completion times of less than 100 s, suggesting inattentive responding; and 9 displayed patterned responses, such as identical answers across items. Consequently, 340 questionnaires were initially deemed valid, yielding an initial valid response rate of 83.95%.

For the LPA and subsequent regression analyses, missing data on the variables included in these analyses were handled using listwise deletion, and no imputation was performed. Specifically, 14 additional cases with missing values on the LPA indicators or regression variables were excluded. Therefore, 326 cases were retained as the final analytic sample. This final analytic sample was within the recommended sample size range for LPA. In the retained three‐profile solution, the smallest profile included 30 participants, accounting for 9.2% of the final analytic sample, which exceeded the recommended minimum proportion of 5% and supported the interpretability of the profile solution.

### 2.4. Data Collection and Informed Consent

The research team leader contacted the directors of the nursing departments to introduce the study and obtain administrative permission and support for participant recruitment. After obtaining administrative permission from the directors of the nursing departments, the research team disseminated the electronic questionnaire to eligible clinical nurses via a secure online survey platform. As many new nurses were rotating across departments and had not yet been assigned to fixed units, the sample was not department‐specific, thereby minimizing potential sampling heterogeneity. The initial page of the electronic questionnaire served as the informed consent form, which comprehensively detailed the study’s objectives, the voluntary nature of participation, the right to withdraw at any time, and assurances of anonymity and confidentiality. Participants were required to carefully review this information and provide their explicit consent by clicking “Confirm to participate in this study” before proceeding to the survey items. Participation was entirely voluntary, and all responses were collected anonymously. The questionnaire was distributed and collected via the online platform Questionnaire Star, which was linked to participants’ WeChat accounts. Each account was permitted to submit the questionnaire only once, thereby preventing duplicate responses. Meanwhile, only anonymized WeChat IDs were visible to the researchers, ensuring participants’ anonymity and helping to reduce common method bias. Upon completion of data collection, all survey responses were securely retrieved and stored by the first author through the designated online survey platform.

### 2.5. Instrument

#### 2.5.1. Demographic Variables

Demographic characteristics, including gender, age, marital status, and educational attainment, were collected as control variables.

#### 2.5.2. Job Crafting

Job crafting was assessed using the 15‐item Job Crafting Questionnaire developed by Slemp and Vella‐Brodrick in 2013 [40]. This scale comprises three dimensions: task crafting, cognitive crafting, and relational crafting. Task crafting refers to employees’ proactive changes to the scope, type, or number of work tasks they perform, with an example item being “I change the scope or types of tasks that I complete at work.” Cognitive crafting reflects employees’ reframing of how they perceive the meaning and value of their work, with an example item being “I remind myself about the significance my work has for the success of the organization.” Relational crafting refers to employees’ proactive changes in work‐related social interactions, with an example item being “I make friends with people at work who have similar skills or interests.”

Participants rated their agreement with each item on a six‐point Likert scale, ranging from 1 = strongly disagree to 6 = strongly agree. Higher scores indicated higher levels of job crafting. The Job Crafting Questionnaire has been widely used in Chinese occupational and nursing samples and has demonstrated satisfactory reliability and construct validity in previous studies [41–45]. In the present study, the Cronbach’s *α* of the total job crafting scale was 0.962, and the Cronbach’s *α* values for the task crafting, cognitive crafting, and relational crafting subscales were 0.901, 0.944, and 0.899, respectively, indicating excellent internal consistency. In the LPA, the mean scores of task crafting, cognitive crafting, and relational crafting were used as the three continuous profile indicators.

#### 2.5.3. Innovative Behavior

Innovative behavior was measured using a 5‐item scale developed by Liu et al., which was based on the original work by Scott and Bruce and Wu et al. [46, 47]. It has also been widely applied in Chinese samples, demonstrating good reliability and validity [48–50]. The scale assesses innovative behavior across idea generation, idea promotion, and idea implementation, which are conceptually consistent with the dimensions of nurse‐specific innovative behavior identified in prior research [51].

### 2.6. Statistical Analysis

SPSS 27.0 and Mplus 8.3 were used for data processing and statistical analysis. Descriptive statistics were used to summarize participants’ sociodemographic characteristics, innovative behavior, and job crafting indicators. Categorical variables were presented as frequencies and percentages. Continuous variables were assessed for normality using the Shapiro–Wilk test; normally distributed variables were presented as mean ± standard deviation, whereas nonnormally distributed variables were expressed as median and interquartile range.

LPA was conducted using Mplus 8.3 to identify subgroups of job crafting among Generation Z new nurses. In this study, the three dimensions of job crafting, namely, task crafting, cognitive crafting, and relational crafting, were included as continuous indicators. Models with one to five latent profiles were estimated and compared. Model fit was assessed using the Akaike Information Criterion (AIC), Bayesian Information Criterion (BIC), sample‐size adjusted Bayesian Information Criterion (aBIC), entropy, the Lo–Mendell–Rubin adjusted likelihood ratio test (LMR), and the bootstrap likelihood ratio test (BLRT) [52]. Lower AIC, BIC, and aBIC values indicated better model fit, whereas higher entropy indicated more accurate classification; entropy values of 0.80 or above were considered acceptable. Significant LMR and BLRT results indicated that a model with k profiles provided a better fit than a model with *k* − 1 profiles. The final model was selected by jointly considering statistical fit indices, classification quality, profile size, theoretical interpretability, and parsimony. Profiles comprising less than 5% of the total sample were considered potentially unstable and were interpreted with caution.

After the optimal latent profile solution was determined, differences in sociodemographic characteristics across profiles were examined using chi‐square tests. Differences in innovative behavior across profiles were examined using one‐way analysis of variance. When the homogeneity of variance assumption was met, least significant difference post hoc tests were used for pairwise comparisons. Before the multinomial logistic regression analysis, multicollinearity among candidate predictors was assessed using variance inflation factors. Multinomial logistic regression was then conducted to examine whether innovative behavior predicted latent profile membership. The low job crafting profile was used as the reference category. Innovative behavior was entered as the main continuous predictor, and age group and marital status were included as categorical covariates based on the univariate results and model stability considerations. Odds ratios (ORs) and 95% confidence intervals (95% CIs) were reported. All statistical tests were two‐sided, and *p* < 0.05 was considered statistically significant.

### 2.7. Ethical Considerations

Ethics approval was obtained from the Ethics Committee of the Xiangya Nursing School of Central South University (Number: E2022110).

### 2.8. Use of AI‐Assisted Tools

During the revision of this manuscript, ChatGPT was used to assist with language polishing, editorial organization, and response drafting. The authors critically reviewed, revised, and verified all AI‐assisted content. The AI tool was not used to generate or analyze data, conduct statistical analyses, or make independent scientific interpretations. The authors take full responsibility for the content of the manuscript.

## 3. Results

### 3.1. Demographic Characteristics of Participants

A total of 326 Generation Z new nurses were included in the final analysis. Among them, 190 participants were female and 136 were male. Regarding age, 92 participants were aged < 20 years, 186 were aged ≥ 20 and < 25 years, and 48 were aged ≥ 25 and < 30 years. For marital status, 174 participants were unmarried and 152 were married. In terms of educational level, 73 participants had an associate degree or below, 232 had a bachelor’s degree, and 21 had a master’s degree or above. The mean innovative behavior score was 27.03 ± 4.55. The demographic characteristics and innovative behavior scores across the three job crafting profiles are presented in Table 1.

**TABLE 1:** Demographic characteristics and innovative behavior across job crafting profiles.

Variables	Total sample (*N* = 326)	Class 1: Low job crafting profile (*n* = 141)	Class 2: Moderate job crafting profile (*n* = 155)	Class 3: High job crafting profile (*n* = 30)	*χ* ^2^/*F*	*p*
Gender, *n* (%)					0.465	0.793
Female	190 (58.3)	80 (56.7)	91 (58.7)	19 (63.3)		
Male	136 (41.7)	61 (43.3)	64 (41.3)	11 (36.7)		
Age, *n* (%)					25.229	< 0.001
< 20	92 (28.2)	52 (36.9)	37 (23.9)	3 (10.0)		
≥ 20, < 25	186 (57.1)	76 (53.9)	95 (61.3)	15 (50.0)		
≥ 25, < 30	48 (14.7)	13 (9.2)	23 (14.8)	12 (40.0)		
Marital status, *n* (%)					34.550	< 0.001
Unmarried	174 (53.4)	98 (69.5)	71 (45.8)	5 (16.7)		
Married	152 (46.6)	43 (30.5)	84 (54.2)	25 (83.3)		
Education, *n* (%)					13.229	0.010
Master’s degree and above	21 (6.4)	11 (7.8)	10 (6.5)	0 (0.0)		
Bachelor’s degree	232 (71.2)	87 (61.7)	119 (76.8)	26 (86.7)		
Associate degree and below	73 (22.4)	43 (30.5)	26 (16.8)	4 (13.3)		
Innovative behavior, mean ± SD	27.03 ± 4.55	23.87 ± 3.68	28.83 ± 3.17	32.57 ± 3.97	117.187	< 0.001

*Note:* Percentages are column percentages. Chi‐square tests were used for categorical variables, and one‐way ANOVA was used for innovative behavior. Class 1 = low job crafting profile; Class 2 = moderate job crafting profile; Class 3 = high job crafting profile.

Abbreviation: SD, standard deviation.

### 3.2. Model Selection

Latent profile models with one to five profiles were estimated using task crafting, cognitive crafting, and relational crafting as continuous indicators. The model fit indices are shown in Table 2. The AIC, BIC, and aBIC decreased from the one‐profile model to the five‐profile model. Entropy values for the two‐ to five‐profile models ranged from 0.864 to 0.971. The LMR and BLRT values were statistically significant for the two‐, three‐, four‐, and five‐profile models.

**TABLE 2:** Fit indices for latent profile models.

Model	AIC	BIC	aBIC	Entropy	Class size, *n* (%)	LMR *p*	BLRT *p*
1‐profile	2701.264	2723.986	2704.954	—	326 (100.0)	—	—
2‐profile	2209.739	2247.608	2215.889	0.971	290 (89.0), 36 (11.0)	< 0.001	< 0.001
3‐profile	1952.137	2005.154	1960.747	0.864	141 (43.3), 155 (47.5), 30 (9.2)	< 0.001	< 0.001
4‐profile	1874.354	1942.518	1885.423	0.893	152 (46.6), 16 (4.9), 136 (41.7), 22 (6.7)	0.030	< 0.001
5‐profile	1801.675	1884.987	1815.204	0.874	86 (26.4), 145 (44.5), 64 (19.6), 16 (4.9), 15 (4.6)	0.002	< 0.001

*Note:* aBIC = sample‐size adjusted Bayesian Information Criterion; LMR = Lo–Mendell–Rubin adjusted likelihood ratio test.

Abbreviations: AIC, Akaike Information Criterion; BIC, Bayesian Information Criterion; BLRT, bootstrap likelihood ratio test.

Although the four‐ and five‐profile models showed lower information criteria than the three‐profile model, they produced small profiles. The four‐profile model included one profile comprising 4.9% of the sample, and the five‐profile model included two profiles comprising 4.9% and 4.6% of the sample. Considering the fit indices, entropy, profile size, parsimony, and interpretability, the three‐profile solution was retained.

### 3.3. Characteristics of the Three Job Crafting Profiles

**FIGURE 1:**
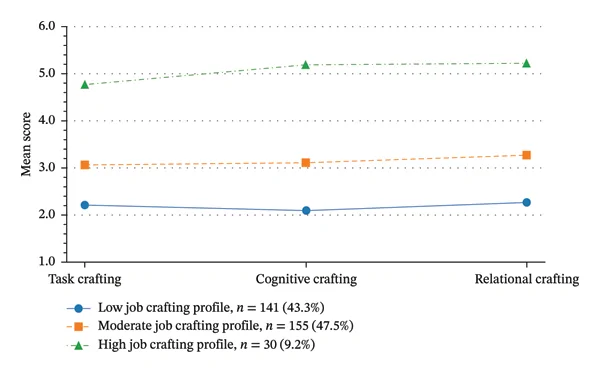
Estimated mean scores of task crafting, cognitive crafting, and relational crafting across the three latent job crafting profiles. Note: Class 1 = low job crafting profile; Class 2 = moderate job crafting profile; Class 3 = high job crafting profile.

The estimated means of the three job crafting dimensions across the three‐profile solution are presented in Table 3 and Figure 1. Class 1 included 141 nurses, accounting for 43.3% of the sample. This class showed the lowest scores across task crafting, cognitive crafting, and relational crafting and was labeled the low job crafting profile.

**TABLE 3:** Estimated means of job crafting dimensions across the three‐profile solution.

Profile	*n* (%)	Task crafting, mean (SE)	Cognitive crafting, mean (SE)	Relational crafting, mean (SE)
Low job crafting profile	141 (43.3)	2.217 (0.051)	2.101 (0.067)	2.272 (0.065)
Moderate job crafting profile	155 (47.5)	3.071 (0.064)	3.110 (0.059)	3.277 (0.060)
High job crafting profile	30 (9.2)	4.771 (0.149)	5.195 (0.146)	5.225 (0.157)

Abbreviation: SE, standard error.

Class 2 included 155 nurses, accounting for 47.5% of the sample. This class showed moderate scores across all three job crafting dimensions and was labeled the moderate job crafting profile.

Class 3 included 30 nurses, accounting for 9.2% of the sample. This class showed the highest scores across task crafting, cognitive crafting, and relational crafting and was labeled the high job crafting profile. Overall, the three profiles showed an ordered low‐to‐high pattern across all job crafting dimensions.

### 3.4. Differences in Demographic Characteristics and Innovative Behavior Across Profiles

Differences in demographic characteristics and innovative behavior across the three job crafting profiles are presented in Table 1. Gender did not differ significantly across the three profiles, χ^2^ = 0.465, *p* = 0.793. Significant differences were found for age, χ^2^ = 25.229, *p* < 0.001, marital status, χ^2^ = 34.550, *p* < 0.001, and educational level, χ^2^ = 13.229, *p* = 0.010. The result for educational level should be interpreted cautiously because one cell had an expected count below 5.

Innovative behavior differed significantly across the three profiles, *F* = 117.187, *p* < 0.001. The mean innovative behavior score was lowest in the low job crafting profile, followed by the moderate job crafting profile, and highest in the high job crafting profile. Post hoc comparisons using LSD showed significant differences between the low and moderate job crafting profiles, mean difference = −4.967, *p* < 0.001; between the low and high job crafting profiles, mean difference = −8.701, *p* < 0.001; and between the moderate and high job crafting profiles, mean difference = −3.734, *p* < 0.001.

### 3.5. Predictors of Job Crafting Profile Membership

Multinomial logistic regression was conducted to examine whether innovative behavior predicted job crafting profile membership, with the low job crafting profile as the reference category. Age group and marital status were included as covariates. Educational level was not entered into the final multinomial logistic regression model because the high job crafting profile contained no participants with a master’s degree or above, resulting in a sparse cell and unstable parameter estimates. The model was statistically significant, χ^2^ = 198.729, df = 8, *p* < 0.001, with Cox and Snell *R* ^2^ = 0.456, Nagelkerke *R* ^2^ = 0.539, and McFadden *R* ^2^ = 0.326. The likelihood ratio tests showed that innovative behavior was a significant predictor of profile membership, χ^2^ = 158.419, df = 2, *p* < 0.001. Detailed regression coefficients are presented in Table 4.

**TABLE 4:** Multinomial logistic regression predicting job crafting profile membership.

Predictor	*B*	SE	Wald	*p*	OR	95% CI
Moderate job crafting profile vs low job crafting profile						
Innovative behavior	0.411	0.052	61.298	< 0.001	1.508	1.360–1.671
Age < 20 vs ≥ 25, < 30	0.661	0.581	1.295	0.255	1.936	0.621–6.041
Age ≥ 20, < 25 vs ≥ 25, < 30	0.339	0.474	0.512	0.474	1.404	0.554–3.557
Unmarried vs married	−0.747	0.361	4.273	0.039	0.474	0.233–0.962
High job crafting profile vs low job crafting profile						
Innovative behavior	0.889	0.115	60.243	< 0.001	2.433	1.944–3.045
Age < 20 vs ≥ 25, < 30	0.422	1.095	0.148	0.700	1.524	0.178–13.040
Age ≥ 20, < 25 vs ≥ 25, < 30	−0.481	0.690	0.486	0.486	0.618	0.160–2.389
Unmarried vs married	−1.370	0.773	3.141	0.076	0.254	0.056–1.156

*Note:* The reference outcome category was the low job crafting profile. Reference categories for predictors were age ≥ 25 and < 30 years and married status. Model fit: χ^2^ = 198.729, *df* = 8, *p* < 0.001; Cox and Snell *R* ^2^ = 0.456; Nagelkerke *R* ^2^ = 0.539; McFadden *R* ^2^ = 0.326.

Abbreviations: CI, confidence interval; OR, odds ratio; SE, standard error.

Compared with nurses in the low job crafting profile, higher innovative behavior was significantly associated with greater odds of membership in the moderate job crafting profile, OR = 1.508, 95% CI 1.360–1.671, *p* < 0.001. Higher innovative behavior was also significantly associated with greater odds of membership in the high job crafting profile, OR = 2.433, 95% CI 1.944–3.045, *p* < 0.001.

## 4. Discussion

This study identified three distinct job crafting profiles among Chinese Generation Z new nurses: the low job crafting profile, the moderate job crafting profile, and the high job crafting profile. Significant differences in age, marital status, educational level, and innovative behavior were found across the three profiles. In the multinomial logistic regression model, innovative behavior remained a significant predictor of membership in profiles with higher levels of job crafting after adjusting for age and marital status.

### 4.1. Demographic Characteristics of Respondents

In this study, nurses aged ≥ 20 and < 25 years accounted for the largest proportion of the sample, reflecting the young age structure of Generation Z new nurses. This finding is consistent with previous evidence showing that new nurses in China are generally concentrated in younger age groups [39]. In terms of educational attainment, the majority of participants held a bachelor’s degree, which differs from the 2023 China Health and Wellness Statistical Yearbook, where the national nursing workforce predominantly comprises individuals with junior college degrees [53]. This may reflect the relatively higher educational requirements and recruitment standards of tertiary hospitals.

The mean innovative behavior score was 27.03 ± 4.55, indicating a moderate level of innovative behavior among Generation Z new nurses. This finding is generally consistent with previous research on innovative behavior among junior clinical nurses [54]. The moderate level observed may be related to limited access to supportive resources, practical opportunities, and organizational environments that encourage innovation. Previous qualitative evidence has shown that inadequate resources and limited managerial support may restrict nurses’ innovation at work [55]. Therefore, further managerial support may be needed to translate Generation Z nurses’ potential for innovation into actual innovative behaviors in clinical practice.

### 4.2. Characteristics of the Three Job Crafting Profiles

The three‐profile solution demonstrated heterogeneity in job crafting among Generation Z new nurses. The low and moderate job crafting profiles together accounted for most of the sample, whereas the high job crafting profile represented a smaller subgroup. This distribution suggests that most new nurses in this sample reported low to moderate levels of job crafting during the early stage of clinical employment.

#### 4.2.1. Characteristics of the Low Job Crafting Profile

The low job crafting profile accounted for 43.3% of the sample and showed the lowest scores across task crafting, cognitive crafting, and relational crafting. This profile may reflect relatively limited engagement in actively adjusting work tasks, reconstructing work meaning, or developing work‐related relationships. For new nurses, low levels of job crafting may be associated with early role transition, limited clinical experience, uncertainty regarding professional responsibilities, and reliance on existing routines or external guidance [56].

At the individual level, new nurses commonly have limited exposure to complex or high‐acuity clinical situations, which may constrain their confidence in adjusting task boundaries or initiating work‐related changes [57]. Previous studies have also suggested that role ambiguity and role conflict may increase dependence on supervisors and limit active exploration among young nurses [58, 59]. In addition, new nurses may focus primarily on acquiring basic clinical skills and meeting immediate work requirements, which may reduce their attention to broader professional role development and meaning reconstruction [60]. Their adaptation to clinical routines, night shifts, responsibilities, and interpersonal relationships may also create pressure during the transition period [61, 62].

At the organizational level, low psychological safety and limited tolerance for errors may constrain nurses’ willingness to try new approaches or speak up about work improvement, thereby limiting proactive behaviors such as job crafting [63, 64]. According to the JD‐R model, employees can increase structural and social job resources through job crafting [65]. However, nurses in the low job crafting profile may have fewer perceived opportunities or resources to engage in such proactive adjustments. Prior nursing management research also suggests that supportive leadership and shared resources are important for promoting job crafting and innovative behavior among nurses [66].

#### 4.2.2. Characteristics of the Moderate Job Crafting Profile

The moderate job crafting profile accounted for 47.5% of the sample and represented the largest group. Nurses in this profile showed moderate levels of task crafting, cognitive crafting, and relational crafting. This suggests that they had begun to make adjustments within their work roles but had not yet reached a high level of job crafting.

Within this profile, task crafting was slightly lower than cognitive and relational crafting. This pattern suggests that these nurses may be more likely to reinterpret the meaning of their work and maintain workplace relationships than to expand or redesign task‐related responsibilities. Compared with the high job crafting profile, the moderate job crafting profile may represent a transitional state in which nurses have adapted to routine clinical work but still require support to broaden task boundaries and participate more actively in work improvement. Previous research has linked limited initiative in expanding work responsibilities to concerns about risk, unfamiliarity with clinical situations, workplace silence, and insufficient professional confidence [67–69].

#### 4.2.3. Characteristics of the High Job Crafting Profile

The high job crafting profile accounted for 9.2% of the sample and showed the highest levels across all three job crafting dimensions. Nurses in this profile had particularly high cognitive and relational crafting scores, together with a high level of task crafting. This suggests that they were more likely to actively interpret the positive meaning of nursing work, develop supportive workplace relationships, and adjust their clinical tasks.

Higher levels of cognitive and relational crafting may be associated with more favorable work‐related outcomes among nurses, including stronger occupational identity, work engagement, and positive work experiences [70, 71]. Although the high job crafting profile represented the smallest subgroup, it may be important for nursing management because these nurses may have greater potential to participate in quality improvement, clinical innovation, peer support, and team development. However, managers should avoid assuming that high job crafting always indicates unlimited capacity. Nurses with high levels of proactive engagement may still require workload monitoring and organizational support to prevent overextension.

### 4.3. Demographic Differences Across Job Crafting Profiles

Age, marital status, and educational level differed significantly across the three profiles in the univariate analyses. The low job crafting profile had a higher proportion of nurses aged < 20 years, whereas the high job crafting profile had a higher proportion of nurses aged ≥ 25 and < 30 years. This may reflect differences in clinical exposure, role familiarity, and professional maturity during the early career stage. Work readiness has been shown to be associated with work‐related outcomes among new graduate nurses [72], and Generation Z nurses’ familiarity with digital tools and receptiveness to new technologies may also influence their work‐related behaviors [11]. However, age was not a significant predictor in the final multinomial logistic regression model after adjustment, suggesting that its role should be interpreted cautiously.

Marital status also differed across profiles. The high job crafting profile had the highest proportion of married nurses. In the multinomial logistic regression model, unmarried nurses had lower odds of belonging to the moderate job crafting profile than to the low job crafting profile compared with married nurses. This result may suggest that marital or family support is associated with more favorable work adjustment for some new nurses. Previous research has emphasized the importance of empowerment and support in the development of newly graduated nurses [73]. Family support may reduce work–family conflict and enhance engagement through both emotional buffering and practical assistance, thereby enabling nurses to invest more effort in work improvement and innovation [74–77]. However, the association between marital status and membership in the high job crafting profile did not reach statistical significance; therefore, this finding should not be overinterpreted.

Educational level differed significantly across profiles in the univariate analysis, but it was not included in the final multinomial logistic regression model because the high job crafting profile contained no participants with a master’s degree or above, resulting in a sparse cell and unstable parameter estimates. Therefore, the relationship between educational level and job crafting profile membership should be interpreted as preliminary. Previous studies have suggested that educational background may be related to nurses’ career orientation, perceived work systems, and work engagement [78, 79]. It is possible that nurses with a bachelor’s degree may show stronger engagement in clinical nursing tasks due to their standardized clinical training and foundational research preparation [79]. In contrast, nurses with a master’s degree or above may have different role expectations or career development trajectories, such as greater involvement in research, advanced training, or nonroutine professional development, which may influence their job crafting behaviors in clinical care [80]. Further studies with larger and more balanced samples are needed to clarify this relationship.

### 4.4. Predictive Effect of Innovative Behavior on Job Crafting Profile Membership

The multinomial logistic regression analysis showed that innovative behavior was a significant predictor of job crafting profile membership. Compared with the low job crafting profile, each one‐unit increase in innovative behavior was associated with a 50.8% increase in the odds of belonging to the moderate job crafting profile and a 143.3% increase in the odds of belonging to the high job crafting profile. This indicates that nurses with higher innovative behavior were more likely to belong to profiles characterized by higher levels of task, cognitive, and relational crafting.

This finding is consistent with previous research showing that innovative behavior is closely related to proactive work behaviors and supportive work environments among nurses [81]. Innovative behavior and job crafting both involve proactive efforts to improve work processes and adapt work roles. Nurses with stronger innovative behavior may be more likely to identify problems in clinical practice, seek new solutions, communicate with colleagues, and adjust task‐related or relational aspects of their work. These behaviors are closely aligned with task crafting, cognitive crafting, and relational crafting [82].

This relationship may also be explained by the proactive nature of innovative behavior. Innovative behavior often involves generating new ideas, seeking support, and implementing changes in work practices [33, 66]. When new nurses engage in innovative behavior in clinical settings, they may also be more likely to take on challenging tasks, develop new skills, and seek support from colleagues and supervisors. These behaviors are consistent with the general concept of job crafting [82]. Previous studies have also suggested that nurses with stronger innovative tendencies or positive psychological resources may demonstrate greater professional confidence and proactive work orientation, which may be associated with higher engagement in job crafting behaviors rather than passive role acceptance [83].

For nursing managers, these findings suggest that cultivating innovative behavior may be an important pathway for promoting job crafting among Generation Z new nurses. Supportive management strategies may include encouraging new nurses to participate in quality improvement activities, providing opportunities for supervised experimentation, recognizing small‐scale innovations in daily clinical work, and creating a psychologically safe environment for proposing new ideas. Such strategies may help new nurses move from low‐ or moderate‐forms of job crafting toward higher levels of proactive work redesign.

## 5. Limitations

This study has some limitations. First, the sample was drawn exclusively from three tertiary general hospitals in Midwestern China using convenience sampling, which may limit the representativeness of the sample. Therefore, the findings may not be fully generalizable to nurses working in other regions, at different levels of hospitals, such as primary or secondary hospitals, or in other healthcare systems. Future research should adopt multicenter designs with more diverse and representative samples to enhance external validity.

Second, all data were obtained through self‐reported questionnaires, which may be subject to response bias. Moreover, as all variables were collected from the same source at a single time point, the results may be affected by potential common method variance. Third, the cross‐sectional design limits the ability to infer causal relationships between innovative behavior and job crafting. Future longitudinal studies are needed to examine the temporal and potentially reciprocal relationships between innovative behavior and job crafting among new nurses.

Finally, educational level was not included in the final multinomial logistic regression model because one education category had no cases in the High job crafting profile, resulting in sparse data and unstable estimates. Future studies with larger and more balanced samples are needed to further examine the role of educational background in job crafting profile membership.

## 6. Conclusions

In this study, three job crafting profiles among Chinese Generation Z new nurses were identified through LPA: the low job crafting profile, the moderate job crafting profile, and the high job crafting profile. Significant differences were observed across these profiles in age, marital status, educational level, and innovative behavior. Multinomial logistic regression further showed that innovative behavior was significantly associated with higher odds of membership in the moderate and high job crafting profiles. These findings highlight the heterogeneity of job crafting among Generation Z new nurses and the importance of innovative behavior in distinguishing different job crafting profiles.

## 7. Implications for Nursing Management

This study highlights the heterogeneity of job crafting among Generation Z new nurses and the key role of innovative behavior. Nursing management strategies should therefore be tailored to different job crafting profiles while also fostering an environment that supports innovation and proactive work adjustment.

For nurses in the Low job crafting profile, management interventions should focus on building professional confidence, psychological safety, and basic role adjustment. Structured orientation programs combined with one‐to‐one mentorship may provide continuous guidance, clarify role expectations, and reduce uncertainty, thereby promoting initial engagement in task crafting. Mentors can also provide timely feedback and demonstrate effective communication and problem‐solving strategies, which may facilitate the gradual development of cognitive and relational crafting. In addition, fostering a psychologically safe environment that tolerates reasonable trial and error may encourage these nurses to gradually participate in new tasks and interpersonal interactions.

For nurses in the moderate job crafting profile, management should focus on strengthening task crafting. Compared with the high job crafting profile, these nurses showed an overall moderate level of job crafting, with task crafting relatively less developed than cognitive and relational crafting. Accordingly, managers should provide opportunities for these nurses to gradually take on additional or more complex clinical tasks within routine practice, while ensuring appropriate guidance and support from senior staff. Head nurses can facilitate this process by offering timely feedback, reinforcing effective task performance, and allowing nurses to participate in patient care improvement, workflow optimization, and team‐based activities. Such strategies may help nurses build on their existing capabilities and progressively expand their task boundaries.

For nurses in the high job crafting profile, management should focus on sustaining and leveraging their proactive behaviors. Providing greater autonomy in workflow organization and supporting their involvement in teaching, research, quality improvement, and clinical innovation activities may enable them to use their strengths more effectively. When appropriate, opportunities to serve as team coordinators or project leaders may help develop their leadership and management potential. At the same time, managers should monitor workload and provide adequate organizational support to prevent overextension and ensure the sustainability of their high level of proactive functioning.

Overall, tailoring management strategies to different job crafting profiles while fostering a culture of innovation may help optimize job crafting, strengthen professional identity, and improve the quality of nursing care.

## Author Contributions

Study conceptualization and design: Bo Zhu, Yang Dong, Boyi Li, Mei Zhou, Mingyu Zhang, Li Xu, Hongtao Guo, and Honghong Wang. Data collection: Bo Zhu, Yang Dong, Boyi Li, Mei Zhou, Mingyu Zhang, and Hongtao Guo. Data analysis: Bo Zhu, Yang Dong, and Boyi Li. study supervision: Honghong Wang, Hongtao Guo, and Bo Zhu. Funding acquisition: Honghong Wang, Mingyu Zhang, and Hongtao Guo. Manuscript writing: Bo Zhu. Critical revisions and review: Bo Zhu, Yang Dong, Boyi Li, Mei Zhou, Mingyu Zhang, Li Xu, Hongtao Guo, and Honghong Wang.

## Funding

This study was supported by the Nursing Society of Inner Mongolia Autonomous Region (Number: NMKY‐202526).

## Disclosure

All authors have read and approved the final version of the manuscript.

## Conflicts of Interest

The authors declare no conflicts of interest.

## Data Availability

The datasets generated and analyzed during the current study are available from the corresponding authors upon reasonable request.
